# The Apoptotic, Angiogenic and Cell Proliferation Genes *CD63*, *S100A6 e GNB2L1* are Altered in Patients with Endometriosis

**DOI:** 10.1055/s-0038-1673364

**Published:** 2018-10

**Authors:** Valéria Aguiar Gomes, Camila de Moraes Bonocher, Júlio César Rosa-e-Silva, Cláudia Cristina Paro de Paz, Rui Alberto Ferriani, Juliana Meola

**Affiliations:** 1 Department of Gynecology and Obstetrics, School of Medicine of Ribeirao Preto, Universidade de São Paulo, Ribeirão Preto, SP, Brazil; 2 Department of Genetics, School of Medicine of Ribeirao Preto, Universidade de São Paulo, Ribeirão Preto, SP, Brazil

**Keywords:** endometriosis, gene expression, molecular genetics, pathophysiology, real-time PCR, endometriose, expressão gênica, genética molecular, fisiopatologia, PCR em tempo real

## Abstract

**Objective** The aim of the present study was to analyze the expression of the *CD63*, *S100A6*, and *GNB2L1*genes, which participate in mechanisms related to the complex pathophysiology of endometriosis.

**Methods** A case-control study was conducted with 40 women who were diagnosed with endometriosis, and 15 fertile and healthy women. Paired samples of eutopic endometrium and endometriotic lesions (peritoneal and ovarian endometriotic implants) were obtained from the women with endometriosis in the proliferative (*n* = 20) or secretory phases (*n* = 20) of the menstrual cycle. As controls, paired endometrial biopsy samples were collected from the healthy women in the proliferative (*n* = 15) and secretory (*n* = 15) phases of the same menstrual cycle. We analyzed the expression levels of the *CD63*, *S100A6*, and *GNB2L1* genes by real-time polymerase chain reaction.

**Results** An increase in *CD63*, *S100A6*, and *GNB2L1* gene transcript levels was observed in the ectopic implants compared with the eutopic endometrium of the women with and without endometriosis, regardless of the phase of the menstrual cycle.

**Conclusion** These findings suggest that the *CD63, S100A6*, and *GNB2L1* genes may be involved in the pathogenesis of endometriosis, since they participate in mechanisms such as inhibition of apoptosis, angiogenesis and cell proliferation, which lead to the loss of cell homeostasis in the ectopic endometrium, thus contributing to the implantation and survival of the tissue in the extrauterine environment.

## Abstract (pt)

**Resumo:**

**Objetivo** O objetivo do presente estudo foi analisar a expressão dos genes *CD63*, *S100A6* e *GNB2L1*, que participam em mecanismos relacionados à complexa fisiopatologia da endometriose.

**Métodos** Um estudo caso-controle foi realizado com 40 mulheres diagnosticadas com endometriose e 15 mulheres férteis e saudáveis. Amostras pareadas de endométrio eutópico e de lesões endometrióticas (implantes endometrióticos peritoneais e ovarianos) foram obtidas de mulheres com endometriose nas fases proliferativa (*n* = 20) ou secretora (*n* = 20) do ciclo menstrual. Como controle, amostras pareadas de biópsia endometrial foram coletadas de mulheres saudáveis nas fases proliferativa (*n* = 15) e secretora (*n* = 15) no mesmo ciclo menstrual. Foram analisados os níveis de expressão dos genes *CD63*, *S100A6* e *GNB2L1* por reação em cadeia da polimerase em tempo real.

**Resultados** Foi observado um aumento nos níveis de transcritos dos genes *CD63*, *S100A6* e *GNB2L1* em implantes ectópicos quando comparado ao endométrio eutópico de mulheres com e sem endometriose, independente da fase do ciclo menstrual.

**Conclusão** Estes achados sugerem que os genes *CD63, S100A6* e *GNB2L1* podem estar envolvidos na patogênese da endometriose, pois participam de mecanismos como inibição de apoptose, angiogênese e proliferação celular, os quais levam à perda da homeostase celular no endométrio ectópico e, portanto, contribuem para o implante e a sobrevivência do tecido no ambiente extrauterino.

## Introduction

Endometriosis is a benign gynecological disease that affects at least 10% of women of reproductive age.^1^ It is defined by the growth of endometrial tissue outside the uterine cavity, which is known as ectopic tissue. Although endometriosis is considered a benign disorder, there are some similarities with cancer; both share invasive potential for the implantation and maintenance of ectopic tissue. Altered immune response, adhesion, invasion, cell proliferation, inhibition of apoptosis, angiogenesis, local estrogen production, and response to injury are important events in the pathogenesis of endometriosis.^2^

Recent studies have demonstrated the molecular differences between the eutopic and the ectopic endometrium, suggesting that distinct patterns of gene expression are involved in the development of endometriosis.^3^ ^4^ ^5^ ^6^ ^7^ ^8^ ^9^ ^10^ The alterations in the expression of certain key genes at specific moments can determine normal and abnormal physiological processes and dysregulate the relevant metabolic pathways influencing the formation of lesions. Therefore, genetic studies are necessary to better understand and define the molecular etiology of this disease.

Previous studies have shown the dysregulation of some genes in endometriosis. These genes are also involved in several pathways, such as the inhibition of apoptosis, cell survival, angiogenesis, and cell proliferation, which suggests their participation in the pathophysiology of endometriosis.^3^ ^4^ In addition, our previous study demonstrated the dysregulation of the *CD63, GNB2L*, and *S100A6* genes in endometriosis. Their involvement in the aforementioned processes and the correlation with cancer suggests that the expression of these genes possibly leads to the establishment and survival of endometriotic implants.^3^ ^4^ Therefore, the evaluation of these genes can clarify some of the mechanisms that underlie the complex pathophysiology of endometriosis.

The purpose of the present study was to compare the expression levels of the *CD63, GNB2L1*, and *S100A6* genes in the endometrial tissue from women without endometriosis, in the eutopic and the ectopic endometrium (pelvic and ovarian endometriotic implants), in the proliferative and secretory phases of the menstrual cycle from the patients with endometriosis.

## Methods

### Ethics, Setting, and Duration

A case-control study was conducted on patients with and without endometriosis in the proliferative and secretory phases of the menstrual cycle. The present study was performed from 2010 to 2012 at the Sector of Human Reproduction from the Department of Gynecology and Obstetrics of Faculdade de Medicina de Ribeirão Preto da Universidade de São Paulo (FMRP-USP), in the state of São Paulo, Brazil. The present study was approved by the Institutional Review Board of the FMRP-USP, protocol HCRP n^o^ 11736/2004, and is linked to a bank of endometriosis tissues, which is also approved, protocol HCRP n^o^ 9699/2006. All subjects (those with endometriosis and the controls) signed a written informed consent to participate in the study. The present work was performed in accordance with the ethical standards of the Declaration of Helsinki.

### Participants and Eligibility Criteria

The women were included if they were 18 to 40 years old, had a body mass index (BMI) < 30 kg/m^2^, were not menopausal, had not been taking any hormonal therapy for at least 3 months before sample collection, had no reproductive disorder or tumor, and had a regular menstrual cycle. Other exclusion criteria included smoking, alcoholism, recreational drug use, any systemic disease (systemic arterial hypertension, diabetes mellitus, immune system diseases, or thyroid diseases).

The patients of the endometriosis group were diagnosed through laparoscopy and histopathological analyses. An experienced surgeon performed the laparoscopy, and the stage of endometriosis was determined according to the classification of the American Society for Reproductive Medicine (1997).^11^

The control group consisted of women of reproductive age (18 to 40 years old), who were submitted to laparoscopy for tubal ligation. These women did not have endometriosis, fibrosis, pelvic adhesion, or infertility.

### Sample Collection and Processing

#### Tissue Samples

The paired tissue samples of the eutopic endometrium and of the endometriotic lesions (peritoneal or ovarian lesions) were collected from 40 women with endometriosis during laparoscopy, and the eutopic endometrial samples were collected using a Euro-Med Novak Endometrial Curette (CooperSurgical, Inc., Trumbull, CT, USA). Only one sample was collected per patient. In the control group, two paired biopsy tissue samples were collected from 15 healthy women according to the phase of the menstrual cycle. The phases of the cycle were determined by histological dating. The samples were stored frozen at - 80° C for later analysis after treatment with RNA*later* Stabilization Solution (Thermo Fischer Scientific, Waltham, MA, USA).

#### Total RNA Extraction

The samples were washed with phosphate-buffered saline (PBS) solution (1x) (8.50 g/L NaCl, 1.11 g/L Na_2_HPO_4_, and 2.81 g/L Na_2_HPO_4_,12H_2_O, 0.20 g/L, and KH_2_PO_4,_ pH 7.0) to remove the RNA*later* solution from the tissues. Next, total RNA (50 mg tissue) was extracted using the TRIzol reagent (Thermo Fischer Scientific, Waltham, MA, USA) according to the manufacturer's instructions. After the treatment of the samples with DNase I (Thermo Fischer Scientific, Waltham, MA, USA), RNA integrity was confirmed by the presence of the ribosomal bands (28S and 18S) after analysis by 1% agarose gel electrophoresis. Total RNA concentrations were determined using a NanoDrop 2000c spectrophotometer (Thermo Fischer Scientific, Waltham, MA, USA) at 260 nm. The RNA was stored at - 80° C until further processing.

#### Relative Quantification by Real-time Polymerase Chain Reaction

An aliquot (1 µg) of total RNA from each sample was reverse-transcribed using random primers from the High Capacity cDNA Archive kit (Thermo Fischer Scientific, Waltham, MA, USA) according to the manufacturer's instructions. The reaction was performed in a Piko Thermal Cycler (Thermo Fischer Scientific, Waltham, MA, USA) for 10 minutes at 25° C; 2 hours at 37° C; 5 minutes at 85° C, and 5 minutes at 4° C.

The relative quantification (RQ) of the expression of the selected genes in the collected samples was performed using an ABI PRISM 7500 FAST equipment (Applied Biosystems, Foster City, CA, USA). The reactions were performed using the TaqMan Gene Expression Assay system, being the TaqMan probe with FAM dye label on the 5′ end and minor groove binder (MGB) and nonfluorescent quencher (NFQ) on the 3′ end. This information is an explanation of which TaqMan Gene Expressed Assay was used. (Applied Biosystems, Foster City, CA, USA). The assay IDs of the probes used were *CD63* (Hs00156390_m1), *GNB2L1* (Hs00272002_m1), *S100A6* (Hs00170953_m1), *GAPDH* (Hs99999905_m1), and *ACTB* (Hs99999903_m1).

Real-time polymerase chain reaction (It’s qRT-PCR. Real-Time Quantitative Reverse Transcription PCR. RT-PCR is Reverse transcription-polymerase chain reaction) was performed in triplicate for each sample using a reaction mixture with a final volume of 20 µL consisting of the following: 10 µL TaqMan Universal PCR Master Mix (2x) (Applied Biosystems, Foster City, CA, USA), 1 µL TaqMan Gene Expression Assay Mix (20x) (Applied Biosystems, Foster City, CA, USA), and 9 µL cDNA diluted 1:50. The reaction conditions were 50° C for 2 minutes, 95° C for10 minutes, 40 cycles of 95° C for 15 seconds and 60° C for 1 minute.

A cDNA pool of the endometrial samples (*n* = 30) obtained from the control group was used as a reference sample (calibrator). The reference genes *GAPDH* and *ACTB* were used for normalizing the reactions. Relative quantification of the analyzed genes was calculated for each sample (control group = 30; and endometriosis group = 40) by the 2^-ΔΔCT^ method.^12^

### Statistical Methods

Statistical analyses were performed with the aid of the SAS 2003 software (SAS Institute Inc., Cary, NC, USA). The RQ values of the genes were log-transformed (log_10_ [2^-ΔΔ*C*T t^ +10]). Logarithmic transformation was necessary, since one of the assumptions (linearity) of the linear model analysis was not satisfied.

The analysis of variance (ANOVA) was performed by including the effects of phases of the menstrual cycle (proliferative and secretory), the type of tissue (peritoneal lesion, endometrioma, eutopic endometrium of patients with and without endometriosis) as well as the interactions among them in the statistical model.

We used the *t*-test for paired samples (eutopic endometrium versus ectopic lesions), and the Welch ANOVA test for unpaired samples (control endometrium versus eutopic endometrium of patients with endometriosis, control endometrium versus lesions of patients with endometriosis, and peritoneal lesions versus ovarian lesions).

**Fig. 1:**
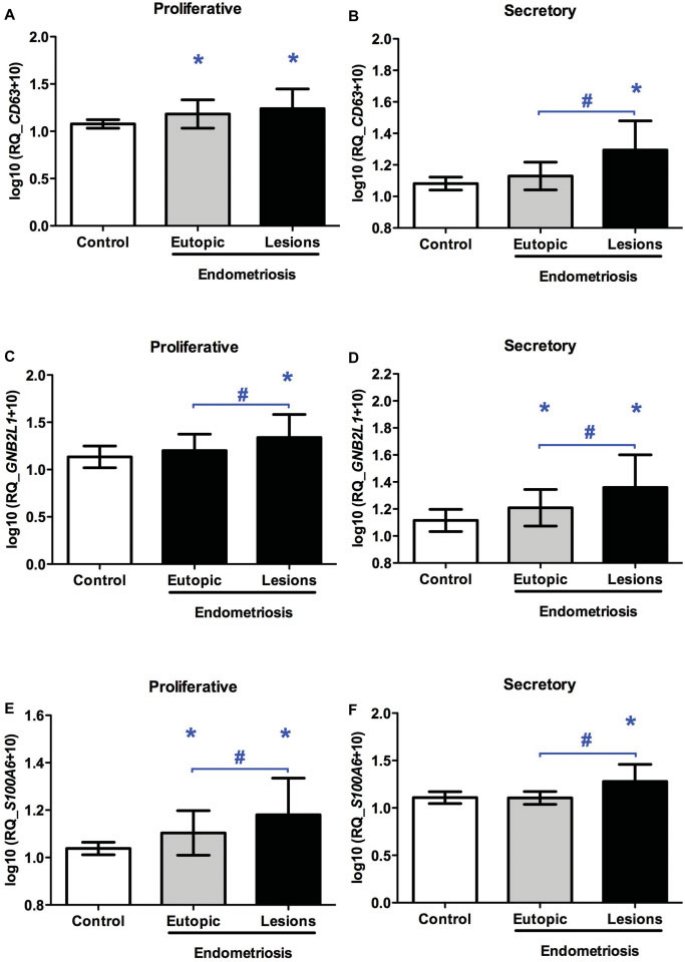
Relative expression of the *CD63, GNB2L1* and *S100A6* genes in human endometrial tissue throughout the menstrual cycle. (**A**) Relative expression of the *CD63* gene in the proliferative phase, (**B**) relative expression of the *CD63* gene in the secretory phase, (**C**) relative expression of the *GNB2L1* gene in the proliferative phase, (**D**) relative expression of the *GNB2L1* gene in the secretory phase, (**E**) relative expression of the *S100A6* gene in the proliferative phase and (**F**) relative expression of the *S100A6* gene in the secretory phase. Control = endometrium of women without endometriosis; Eutopic = endometrium of women with endometriosis; Lesions = endometriotic lesions (peritoneal and ovarian lesions). *p < 0.05 versus control group (unpaired analyses). #*p* < 0.05 versus eutopic group (paired analyses). Data are shown as mean ± standard deviation.

Data, which are log_10_ (2^-ΔΔ*C*T^ +10)-transformed, are illustrated in Fig. 1 with mean and standard deviation (SD). The analyses were satisfactory, with a test power (1-β) of at least 80%, and the results were considered statistically significant when *p* < 0.05.

## Results

There was no significant difference between the endometriosis and control groups regarding mean age and standard deviation (33.87 ± 2.69 and 33.5 ± 4.37 years old, respectively). The control group consisted of 15 women of reproductive age. Fifteen biopsy samples were obtained during the proliferative phase, and 15 during the secretory phase of the menstrual cycle. The endometriosis group consisted of 40 women, and of these patients, 21 were attended to due to infertility, and 19 due to pelvic pain at the outpatient clinics of marital infertility, and pelvic pain and endoscopy of the university hospital of the FMRP-USP. The number of peritoneal lesions studied in the patients classified as stage I (*n* = 6; in the secretory phase), stage II (*n* = 11; 7 in the proliferative phase and 4 in the secretory phase), stage III (*n* = 2; in the proliferative phase), and stage IV (*n* = 1; in the proliferative phase) was 20, and that of the ovarian endometrioma lesions studied in the patients classified as stage III (*n* = 8; 4 in the proliferative phase and 4 in the secretory phase) and stage IV (*n* = 12; 6 in the proliferative phase and 6 in the secretory phase) was 20.

The results of gene expression in the analyzed tissues are illustrated in Fig. 1. There was no difference in the expression of target genes between the peritoneal and ovarian lesions (data not shown). Thus, the peritoneal and endometrioma lesions were pooled for the remaining analyses.

Increased expression of the *CD63*, *GNB2L1*, and *S100A6* genes was found in the endometriotic lesions regardless of the phases of the menstrual cycle that were studied (Fig. 1). Furthermore, increased expression of the *CD63*, and of the *S100A6* genes (in the proliferative phase), and of the *GNB2L1* gene (in the secretory phase) was detected in the eutopic endometrium of the patients of the endometriosis group compared with the control endometrium (Figs. 1A, E and D; respectively). However, when the proliferative and secretory phases were compared with each other in the endometrial control (*CD63* *p* = 0.52; *GNBL1* *p* = 0.79; *S100A6* *p* = 0.052), eutopic endometrium (*CD63* *p* = 0.38; *GNBL1* *p* = 0.41; *S100A6* *p* = 0.44) and endometriotic lesions (*CD63* *p* = 0.94; *GNBL1* *p* = 0.49; *S100A6* *p* = 0.24), no difference was observed.

## Discussion

The present study identified the increased expression of the *CD63*, *GNB2L1*, and *S100A6* genes in the ectopic lesions of women with endometriosis compared with the endometrium of the control group during both the proliferative and secretory phases of the menstrual cycle. In addition, the expression of these genes had increased in the lesions compared with the eutopic endometrium of the same endometriosis patient during the secretory phase of the menstrual cycle. However, when the eutopic endometrium of the women with endometriosis were compared with the endometrium of the control women, the expression of the *CD63* and of the *S100A6* genes had increased only in the proliferative phase in the endometrium of the women with endometriosis, and the increased expression of the *GNB2L1* gene was observed only in the secretory phase.

These results are in agreement with our previously obtained results, which showed the increased expression of the target genes in endometriotic lesions in a large-scale hybridization study based on subtractive libraries.^3^ However, in the present study, we used RT-PCR to quantify and confirm the dysregulated expression of these genes during the phases of the menstrual cycle. This technique has been defined as the gold standard for transcript quantification due to its high sensitivity and good reproducibility. In addition, possible false-positive results can be revealed when this technique is used for the validation of screening methodologies.^13^ The obtained results confirmed the increased expression of these genes in endometriotic lesions.

One of the possible explanations for the differences observed between the eutopic and the ectopic endometrium of the patients with endometriosis is that endometriosis is a multifactorial disorder, which is influenced by genetic and environmental factors. Studies have shown that different endocrine environments, such as the peritoneal fluid and the intraovarian microenvironment of the lesions, are critical to endometrial implantation, suggesting that the interaction between these different endocrine environments and the ectopic endometrium is important in the pathophysiology of endometriosis.^14^

In the present study, we have detected the increased expression of the *CD63* gene in endometrial lesions compared with the eutopic tissue of women with and without endometriosis. The human *CD63* gene, which codes for a tetraspanin, has been mapped to the chromosome region 12q13, and was first discovered as a surface antigen that is abundantly expressed in cells in the initial stage of melanoma and on the surface of activated blood platelets.^15^ The *CD63* gene interacts directly and indirectly with various molecules, such as integrins, other tetraspanins, cell surface receptors, kinases, protein adaptors, and other proteins, including the L6 antigen, syntenin-1, TIMP-1, and MT1-MMP.^16^ ^17^ ^18^ ^19^ ^20^ ^21^ ^22^ ^23^ ^24^ ^25^ ^26^ ^27^ The *CD63* gene was recently identified to bind to the TIMP-1 protein, which is known to be involved in several cellular processes and in tumor development.^26^ The complex of *CD63*, TIMP-1 and β1 integrin mediates the activation of cell survival pathways through the activation of the focal adhesion kinase (FAK), as well as of the PI3-K and extracellular signal–regulated kinase (ERK) pathways, and to the inhibition of apoptosis.^26^ ^28^ ^29^ Therefore, we hypothesized that the increase in the expression of the *CD63* gene may be related to the promotion of the survival of ectopic endometrial cells, thus favoring the development of endometriotic lesions.

Another important gene that was suggested by Meola et al^3^ and Dentillo et al^4^ to be involved in the pathophysiology of endometriosis is *GNB2L1*, which is commonly called *RACK1* (receptor for activated C-kinase 1) and is located in the chromosome region 5q35.3. It possesses 7 WD domains (tryptophan-aspartate), which provides *RACK1* with the potential to act as an adaptor or scaffold protein in the interactions with various molecules.^3^ ^4^ ^30^ Many cellular functions are attributed to *RACK1*, such as cell growth, chemotactic adhesion and migration, which are mediated by the interaction with integrin and Src, as well as the suppression of apoptosis, anti-inflammation and angiogenesis.^31^ ^32^ ^33^ ^34^ ^35^

In our study, the expression of the *GNB2L1* gene was considerably higher in endometriotic lesions than in the eutopic endometria of women with and without endometriosis. Studies have indicated that the overexpression of this gene positively regulates cell adhesion and migration.^36^ The increased expression of the *GNB2L1*gene and its interaction with IGF-IR augmented the mobility of transformed MCF-7 cells, showing that this gene promotes the anchorage-independent growth of ovarian tumor cells via the insulin growth factor 1R (IGF-1R)/STAT3 pathway, whereas other authors had reported discordant results when analyzing the Src pathway.^37^ ^38^ ^39^ ^40^ Moreover, the *GNB2L1* gene is also overexpressed during angiogenesis, and in breast and colon tumors, non-small cell lung carcinoma, oral squamous cell carcinoma, and melanoma.^31^ ^41^ ^42^ ^43^ ^44^ Thus, the upregulation of the expression of the *GNB2L1* gene suggests its participation in processes such as angiogenesis, suppression of apoptosis, and cell proliferation, which are the steps involved in the pathology of endometriosis.

The third gene evaluated was *S100A6*, whose expression had increased considerably in endometriotic lesions compared with the eutopic endometrium of women with and without endometriosis. The *S100A6* gene*,* whose protein product is also known as calcyclin, has Ca^2+^-binding sites.^45^ Some of the functions associated with this protein and changes in the expression of this gene include cytoskeleton dynamics, cell cycle, apoptosis, overexpression in cells with proliferative potential, and increased induction of cell proliferation.^46^ ^47^ ^48^ ^49^ ^50^ ^51^

Liu et al^52^ showed that the overexpression of the *S100A6* gene in endometrial stromal cells promoted the upregulation of β-catenin expression. The β-catenin is an essential component of the Wnt/β-catenin signaling pathway, which has been demonstrated to be activated in the mid-secretory endometrium of infertile patients with endometriosis.^53^ This pathway is involved in the control of proliferation, migration, and invasion, which are important steps in the pathogenesis of endometriosis. Recently, Zhang et al^54^ demonstrated that inhibition of the expression of the *S100A6* gene significantly reduced the migratory ability of eutopic endometrial stromal cells, induced their apoptosis, and had antiproliferative effect. In addition, both increased protein levels and increased transcript levels of the *S100A6* gene have been observed in many types of tumors.^54^

The increased protein levels of the *S100A6* gene promote cell apoptosis under oxidative stress.^55^ However, the proapoptotic function of this gene is contradictory, as reduced expression of this gene has been observed during the apoptosis of human breast cancer cells, and the expression of the *S100A6* gene has also been demonstrated to inhibit the apoptosis of cardiac myocytes.^56^ ^57^ An increased expression of this gene increases cell adhesion and inhibition of cell invasion, and promotes mobility in a way that is yet to be understood.^58^ ^59^ However, when its expression is insufficient or is negatively regulated, this gene inhibits the proliferation of fibroblasts, osteoblasts, and pancreatic tumor cells.^46^ ^60^ ^61^ Since the *S100A6* gene is involved in cell cycle progression, cell differentiation, the interactions with the cytoskeleton, and cell proliferation, it is possible that the changes in the expression of this gene that were observed in the present study could be implicated in the development of endometriosis.

No differences in the levels of expression of the studied genes were observed when the proliferative and secretory phases were compared with each other in the endometrial control, in eutopic endometria and in endometriotic lesions. However, the eutopic endometria of women with endometriosis had an increased expression of the *CD63* and *S100A6* genes in the proliferative phase, while the increased expression of the *GNB2L1* gene was observed in the secretory phase when compared with the control group; these results could be explained by the changes related to the disease. Although some authors relate the changes of gene expression with hormonal fluctuations, there are few studies and there are controversies in the literature regarding the genes studied. Okada et al^62^ showed that the mRNA levels of the *CD63* gene were substantially reduced during the secretory phase of the menstrual cycle compared with the proliferative phase, and that the expression of the mRNAs is negatively regulated by progesterone, thus associating this gene with functions such as proliferation and differentiation of the endometrium (decidualization). In contrast, Brar et al^63^ found no change in the expression of this gene during decidualization. Tong et al^64^ studied the expression of the *S100A6* gene in the human endometrium throughout the menstrual cycle, and no change was detected.

A limitation of our study was the small sample evaluated due to the restrictive eligibility criteria adopted, limiting the generalization of the study.

## Conclusion

Our results suggest that the altered expression of the *CD63, S100A6*, and *GNB2L1* genes may be involved in the pathophysiology of endometriosis. This is because the CD63, RACK1 and calcyclin proteins participate in the inhibition of apoptosis, angiogenesis and cell proliferation, which can lead to the loss of cell homeostasis in the ectopic endometrium. Future studies are needed to assess the function of the identified genes, and to characterize their roles in the development of the disease. Another aspect that should be addressed is that our study was based on the premise that tissue invasion occurs due to the reflux of viable endometrial cells. Although this idea is still one of the most accepted today, it is only a hypothesis that has not been confirmed.
